# Early-Life Neglect Alters Emotional and Cognitive Behavior in a Sex-Dependent Manner and Reduces Glutamatergic Neuronal Excitability in the Prefrontal Cortex

**DOI:** 10.3389/fpsyt.2020.572224

**Published:** 2021-01-26

**Authors:** Xiuping Sun, Yu Zhang, Xianglei Li, Xinmin Liu, Chuan Qin

**Affiliations:** ^1^National Health Commission Key Laboratory of Human Disease Comparative Medicine, Beijing Engineering Research Center for Experimental Animal Models of Human Critical Diseases, Institute of Laboratory Animal Science, Chinese Academy of Medical Science (CAMS) & Peking Union Medical College (PUMC), Beijing, China; ^2^Peking Union Medical College, Institute of Medicinal Plant Development, Chinese Academy of Medical Science, Beijing, China

**Keywords:** child neglect, maternal separation, learning and memory, anxiety like behavior, neuronal excitability, rats

## Abstract

Early-life neglect in critical developmental periods has been associated with emotional and cognitive consequences. Maternal separation (MS) has been commonly used as a rodent model to identify the developmental effects of child neglect. However, reports have shown considerable variability in behavioral results from MS studies in both mice and rats. Difficulties in developing reliable child neglect models have impeded advances in identifying the effects of early-life stress. Accumulating evidence shows that neuronal intrinsic excitability plays an important role in information processing and storage in the brain. The prefrontal cortex (PFC) integrates information from many cortical and subcortical structures. No studies to date have examined the impact of early-life stress on glutamatergic neuronal excitability in the PFC. This study aimed to develop a reliable child neglect rat model and observe glutamatergic neuronal excitability in the PFC. An MS with early weaning (MSEW) rat model was developed. Rats were separated from the dam for 4 h per day on postnatal days (PNDs) 2–5 and for 8 h per day on PNDs 6–16 and then weaned on PND 17. A battery of behavioral tests was used to assess anxiety-like behavior, coping behavior, working memory, spatial reference memory, and fear memory. The action potentials (APs) of glutamatergic neuronal membranes were recorded. MSEW resulted in anxiety-like behavior, a passive coping strategy and increased fear memory in male rats and decreased locomotor activity in both sexes. MSEW slightly impaired working memory during non-stressful situations in female rats but did not change spatial reference memory or associative learning under stressful circumstances in either sex. MSEW reduced the number of glutamatergic neuron APs in male rats. Our findings showed that MS with early weaning induced anxiety-like behavior in male rats. The reduced glutamatergic neuronal excitability may be associated with the emotional alteration induced by MSEW in male rats. In addition, MSEW induced adaptive modification, which depended on a non-stressful context.

## Introduction

Child neglect is the most common form of early-life stress in both Western and Eastern countries (1–3). The number of left-behind children has been increasing dramatically in recent decades in China (4). The developing brain is particularly sensitive to early-life neglect. It is well-known that child neglect increases the risk for the development of many psychiatric disorders, including anxiety disorder, post-traumatic stress disorder (PTSD), and depression (5, 6). Considering the importance of mother–infant attachment in the early-life period, maternal separation (MS) is one of the most widely used models to elucidate the effects and neurobiological mechanisms of child neglect (7). However, due to the different MS paradigms and strains, the findings of the animal behavioral changes in both mice and rats are inconsistent. Some studies have reported that MS induces impaired memory (8), anxiety, and depressive behavior in rodents (9, 10). However, other studies observed paradoxical behavioral effects (11). Difficulties in developing reliable child neglect models have impeded advances in identifying early-life stress effects. The two- or three-hit stress model, which often induces a consistent behavioral phenotype, has been increasingly studied in recent years (12). Early weaning is another early-life neglect model used to replicate early-life adversities (13, 14). George et al. (15) developed a “two-hit” model that combined MS with early weaning (MSEW), which also elicited inconsistent behavioral outcomes in mice (15–17). The brain development of rats is different from that of mice, and the MS rat model seems to elicit more consistent stress-related alterations than that of mice (18). For example, MS causes a significant increase in defensive-exploratory behavior, a relatively conserved circuit between humans and rodents, in rats but not in mice (19). In the current study, we developed an MSEW rat model to observe long-lasting behavioral changes and provided a reliable animal model of child neglect.

Accumulating evidence shows that neuronal intrinsic excitability plays an important role in information processing and storage in the brain (20). A previous study reported the effect of stress on neural intrinsic excitability in the amygdala, which is involved in governing stress responsivity (21, 22). The prefrontal cortex (PFC) integrates information from many cortical and subcortical structures, including the ventral hippocampus, amygdala, and mediodorsal thalamus. There is growing evidence that the PFC is involved in the generation and regulation of complex cognitive functions such as emotional regulation, working memory, decision-making, planning, and reasoning (23). It is important to understand how early-life stress can influence neural excitability. Until now, the effect of early-life stress on neuronal excitability in the PFC has remained unclear. Here, we explored the possible influence of MSEW on glutamatergic neuronal excitability in the PFC in rats.

## Materials and Methods

### Animals

Adult male and female Sprague–Dawley (SD) rats were purchased from Beijing HFK Bioscience Co., Ltd., and habituated to the animal facilities for 2 weeks. For breeding, a single male rat and two female rats were group-housed in cages for 10 days. Females were housed individually in the last week of pregnancy and observed for births, denoted as postnatal day (PND) 0. All animals were maintained under standard conditions (22–23°C with a 12/12-h light/dark cycle) and received standard food and tap water *ad-libitum*.

### Maternal Separation With Early Weaning Protocol

The protocal was performed that described by George et al. (15). Entire litters were randomized to the control group (male, eight per group; female, 13 per group) or the MSEW group (male, nine per group; female, 13 per group) on PND 0. Control animals were left undisturbed and weaned at PND 21. The MSEW procedure was conducted as described previously (16). MSEW animals were separated from the dam for 4 h per day on PNDs 2–5 and for 8 h per day on PNDs 6–16 and then weaned on PND 17. Litters were placed individually in a separate room on a heating pad (32°C). After weaning, animals were group-housed with littermates of the same sex.

### Behavioral Experiments

At the age of 3 months, the male and female offspring underwent a battery of behavioral tests. All experiments were conducted during the light phase of the cycle and in a temperature-controlled room (22–23°C, humidity was between 40 and 70%) with low-light intensity (10 lux). On the day of the experiment, rats were moved to the testing room, where they remained in their home cage for a 60-min acclimation period. The tests were completed in the order of the least to the most stressful. The open field test, novel object recognition (NOR) test, elevated plus maze test, Y maze, and Barnes maze were conducted using EthoVision video tracking software. Tests were conducted in the following order: (1) open field; (2) NOR test; (3) elevated plus maze; (4) Y maze; (5) Barnes maze; (6) forced swim test; and (7) fear conditioning. Testing areas were thoroughly cleaned with 75% alcohol solution between trials to remove any olfactory traces.

### Open Field Test

The open field test was used to study spontaneous locomotor activity and anxiety behavior. The experimenters were blinded to the treatment of the rats. The open field (80 × 80 × 50 cm) was divided into a central zone and the surrounding border zone (15 cm from the wall). The rats were gently placed in the open field for 5 min of testing. The total distance moved, the distance moved in the border area, time in the center area, velocity, and number of fecal droppings were recorded and analyzed to evaluate locomotor activity and anxiety-related behavior. Thigmotaxis was assessed by the ratio of the distance moved in the border area to the total distance moved, expressed as a percentage.

### Elevated Plus Maze Test

The elevated plus maze test was used to assess animal anxiety and exploratory behavior. The maze comprised two open arms (50 × 10 cm) and two closed arms (50 × 10 × 30 cm) with a central platform (10 × 10 cm). The maze was elevated 70 cm above the floor. The rat was placed in the central platform with its head facing an open arm for a 5-min test. The time spent on the open and closed arms, center area, and the distance moved were recorded. The head-dipping frequency was scored from the video by a trained observer who was blinded to the treatment of the rats. The percentage of time spent on the open arms (%OT = 100 × time spent on open arms/(time spent on open arms + time spent on closed arms) was calculated.

### Novel Object Recognition Test

The NOR test was used here to study non-spatial memory. The test was performed in the same arena employed for the open field test. For habituation, the rat was placed into the open field without stimuli for 10 min for 2 days. For training, two identical objects were placed near the two corners of the open field (15 cm from each adjacent wall). The rat was placed into the center of the open field facing the opposite wall and was allowed 5 min for free exploration of the arena. The test began after a delay of 2 h, with the same object that was used in the training phase (familiar object) and a novel object set into the arena. The rat was placed into the arena and allowed to explore the objects for 3 min. The positions of the objects in the test and the objects used as novel or familiar were counterbalanced between the animals. The time that each rat interacted with the familiar object and the novel object was recorded. The total exploration time (e1) was calculated during the training session for two identical objects. The discrimination index (d1) was calculated as the time spent exploring the novel object minus the time spent exploring the familiar object. The discrimination ratio was calculated as the time spent exploring the novel object minus the time spent exploring the familiar object divided by the total exploration time.

### Y Maze Novel Preference Arm Test

The Y maze novel preference arm test was used to study spatial working memory.

The Y maze consisted of three identical arms (50 × 10 × 30 cm) diverging at a 120° angle from one another. The rat was placed inside the start arm of the Y maze while the novel arm was closed. Then, the rat was allowed to explore the start and other arms but not the novel arm during the 5-min trial. After a 15-min delay, the rat was placed in the start arm and was allowed to explore all three arms for 5 min. The number of entries made into each arm was counted to determine spatial working memory. The percentage of entries into the novel or other arm was calculated as the number of entries into the novel or other arm divided by total entries into the three arms.

### Barnes Maze Test

The Barnes maze test was used to assess hippocampus-dependent spatial learning and memory. The maze was a circular open platform (diameter: 120 cm, height: 65 cm) with 12 equally spaced holes (diameter: 10 cm) around the edges. The escape box (l × w × h: 25 × 6 × 6 cm) was under one of the holes. On the habituation day, the rat was placed under a start box in the center of the circular platform for 10 s. Then, the start box was removed, the buzzer was turned on, and the rat was motivated to escape and gently guided to the hole connected to the escape box (target hole). Immediately after the rat entered the escape box, the buzzer was turned off. Each rat was allowed to remain in the escape box for 1 min, removed, and then returned to the home cage. In the training phase, the rat was placed in the center of the platform and allowed to explore the maze for 3 min. The training tests consisted of three trials for 3 days. In the probe test, 24 h after the last training test, the escape box was removed. Each rat was given 3 min to explore the maze. Escape latency, distance, and velocity were recorded for later analysis.

### Forced Swim Test

Immobility in the forced swim test is interpreted as an inability to actively cope with an aversive situation, and high immobility is believed to reflect increased depressive behavior. On the 1st day, rats were individually placed in a cylinder (diameter: 22 cm; height: 45 cm) filled with water (25°C, depth: 35 cm) for 15 min. Twenty-four hours later, rats were placed in the same cylinder again for 5 min. The climbing time and immobility time were recorded by a trained observer who was blinded to the treatment of the rats. Rats were dried and returned to their home cages after the test. Immobility was defined as the animal floating and making minimal movements necessary to keep its head above water. Climbing behavior was defined as upward-directed movement of the forepaws.

### Contextual Fear Conditioning Test

Contextual fear conditioning was performed to assess hippocampus-dependent associative learning. For habituation, the rat was placed into the conditioning chamber without stimuli for 5 min. In the training phase, the rat was placed into the conditioning chamber (Med Associates, USA) for 3 min and then received five shock and tone pairs (30-s tone; 5 kHz; 70 dB; 1-s foot shock; 0.65 mA DC current) at an interval of 30 s. Contextual fear conditioning was measured 24 h after the training phase in the same chambers. The rat was placed into the same chamber, and no shock or tone was delivered. Freezing behavior was recorded for 5 min with specialized software (Video Freeze, Med Associates, USA).

### Acute Slice Preparation and Electrophysiological Recording

#### Slice Preparation

At 3 days following behavioral testing, rats (*n* = 3–5 per group) were sacrificed under deep pentobarbital sodium anesthesia [50 mg/kg body weight, intraperitoneally (i.p.)], and their whole brains were rapidly dissected and submerged in ice-cold, oxygenated (95% O_2_, 5% CO_2_) cutting solution containing (in mM) 2.5 KCl, 1.25 NaH_2_PO_4_, 0.5 CaCl_2_, 10 MgSO_4_, 26 NaHCO_3_, 10 glucose, and 230 sucrose, pH 7.4, 300–310 mOsm. Coronal slices (250 μm) containing the medial PFC were cut with a Leica VT1000S vibrating microtome (Leica Instruments, Germany) and transferred to an incubation chamber with oxygenated, warm (32°C) regular artificial cerebrospinal fluid (ACSF) containing (in mM) 126 NaCl, 2.5 KCl, 1.3 MgCl_2_, 1.2 NaH_2_PO_4_, 2.4 CaCl_2_, 18 NaHCO_3_, and 10 glucose, pH 7.4, 290–300 mOsm. Slices were then allowed to equilibrate for ~1 h at room temperature.

#### Whole-Cell Recording

The excitability of glutamatergic neurons in the PFC was assessed following MSEW stress by recording the action potentials (APs) of glutamatergic neuronal membranes. After the recovery period, individual slices were placed in the submerged recording chamber, and the tissue was continuously perfused (2 ml/min) with ACSF. The recording chamber was placed on the fixed stage of an Olympus BX51 microscope (Olympus, Germany) equipped with video-enhanced infrared differential interference contrast. Whole-cell recordings were obtained from cortical neurons of medial PFC layer II/III. The patch pipettes were pulled from borosilicate glass capillary tubes (Sutter 150-86-10, USA) using a PC-10 pipette puller (Narishige, Japan). The resistance of pipettes varied between 5 and 8 MΩ when filled with a K^+^ Met sulfonate intracellular solution containing (in mM) 140.5 K+ Met sulfonate, 7.5 NaCl, 10 4-(2-hydroxyethyl)-1-piperazineethanesulfonic acid (HEPES) hemisodium salt, 2 Mg_ATP, and 0.2 Na_GTP, pH 7.33, 300–310 mOsm. Data were recorded using a Multiclamp 700B amplifier and a Digidata 1440A interface controlled by Clampex 10.6 (Molecular Devices, USA). Signals were digitized at 20 kHz and low-pass filtered at 10 kHz. Series resistance was on the order of 10–30 MΩ and was approximately 60%−80% compensated. Data were discarded when the series resistance increased or decreased by more than 20% during the course of the recordings. Input resistance resulted from ΔV divided by the injection of unit current (i.e., 100pA). Afterhyperpolarization (AHP) was measured as previously described (24).

In the PFC, the threshold, amplitude, and half spike time of the glutamatergic neuron single APs were recorded. The number of APs obtained in response to a series of 600-ms current steps from 0 to +500 pA with increments of 100 pA at the fixed potential of −80 mV was obtained. Glutamatergic neurons were distinguished as described previously (25).

### Statistical Analysis

SPSS 20 was used for statistical analysis. All results are presented as the mean ± standard error of the mean (SEM). Data were analyzed using two-way analysis of variance (ANOVA) (sex × MSEW). A repeated-measure ANOVA was performed to assess Barnes maze test training. Student's *t*-test was used when comparing only two groups on one behavioral measure. The normality and homogeneity of variances were tested using the one-sample Kolmogorov–Smirnov test (*P* > 0.05) test and Levene's test (*P* > 0.05). When the assumption of the normality and homogeneity of variances was not met, non-parametric tests (Kruskal–Wallis and Mann–Whitney U) were used to detect significant differences. In the Y maze, paired Student's *t*-test was used to determine whether the percentage of entries into the novel arm differed from the percentage of entries into the other arm. *P* < 0.05 was considered statistically significant for all data. Graphs were prepared using GraphPad Prism 5 (GraphPad Software, United States).

## Results

### Effect of Maternal Separation With Early Weaning on Anxiety-Like Behavior and Locomotor Activity

#### Open Field Test

**Figure 1:**
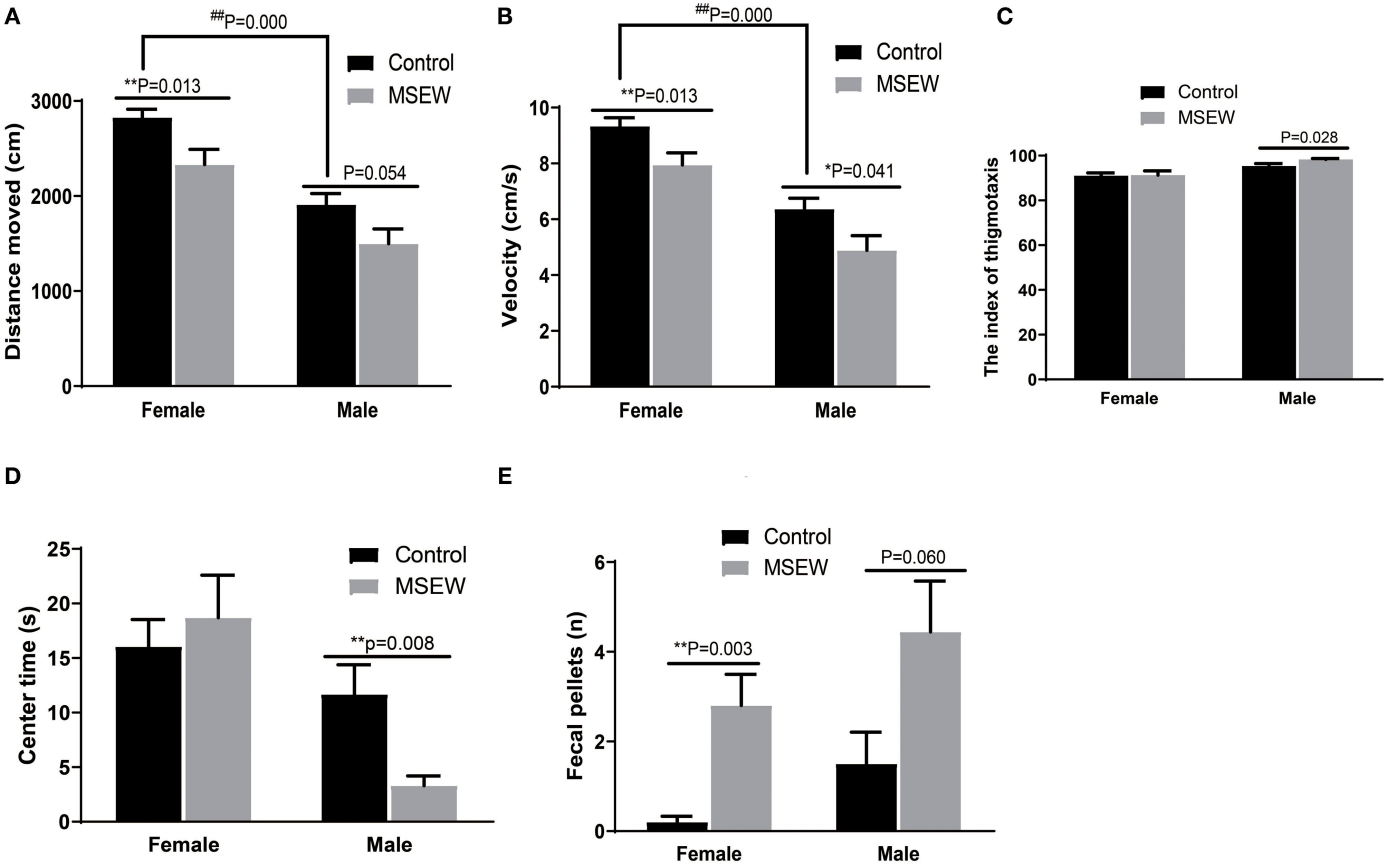
Maternal separation with early weaning (MSEW) induced anxiety-like behavior in the open field test in male rats and decreased locomotor activity in both males and females. MSEW significantly reduced the distance moved and velocity in the open field in both female and male rats **(A,B)**. MSEW increased the index of thigmotaxis in male rats and reduced the center time only in males **(C,D)**. Female and male MSEW rats had increased numbers of feces. **(E)** All results are presented as the mean ± standard error of the mean (SEM) of *n* = 8–10 rats per group. **P < 0.01, control vs. MSEW of the same sex; ^##^*P* < 0.01, male vs. female in controls.

There was no interaction of sex and MSEW in the distance moved [*F*_(3.33)_ = 0.106, *P* = 0.747], velocity [*F*_(3.33)_ = 0.043, *P* = 0.838], or the index of thigmotaxis [*F*_(3.33)_ = 0.931, *P* = 0.342]. Significant main effects of MSEW and sex were found on the distance moved [*F*_(3.33)_ = 11.610, *P* = 0.002; *F*_(3.33)_ = 43.029, *P* = 0.000] and velocity [*F*_(3.33)_ = 12.347, *P* = 0.001; *F*_(3.33)_ = 44.293, *P* = 0.000]. MSEW significantly reduced the distance moved and velocity in both sexes (Figures 1A,B). Control female rats were more active in the distance moved and velocity than control male rats. MSEW increased the index of thigmotaxis in male rats (Figure 1C). There was no interaction of sex and MSEW in the center time in the open field [*F*_(3.33)_ = 2.112, *P* = 0.156]. A significant main effect of sex was found on the center time [*F*_(3.33)_ = 8.859, *P* = 0.005]. MSEW reduced the center time in males (Figure 1D). There was no interaction of sex and MSEW in the number of feces [F_(3.33)_ = 0.054, *P* = 0.817]. A significant main effect of MSEW was found in the number of feces [*F*_(3.33)_ = 14.062, *P* = 0.001]. The assumption of normality for the number of feces was not met (*P* = 0.042), and a Mann–Whitney test was conducted. MSEW increased the number of feces in both sexes (Figure 1E).

#### Elevated Plus Maze Test

**Figure 2:**
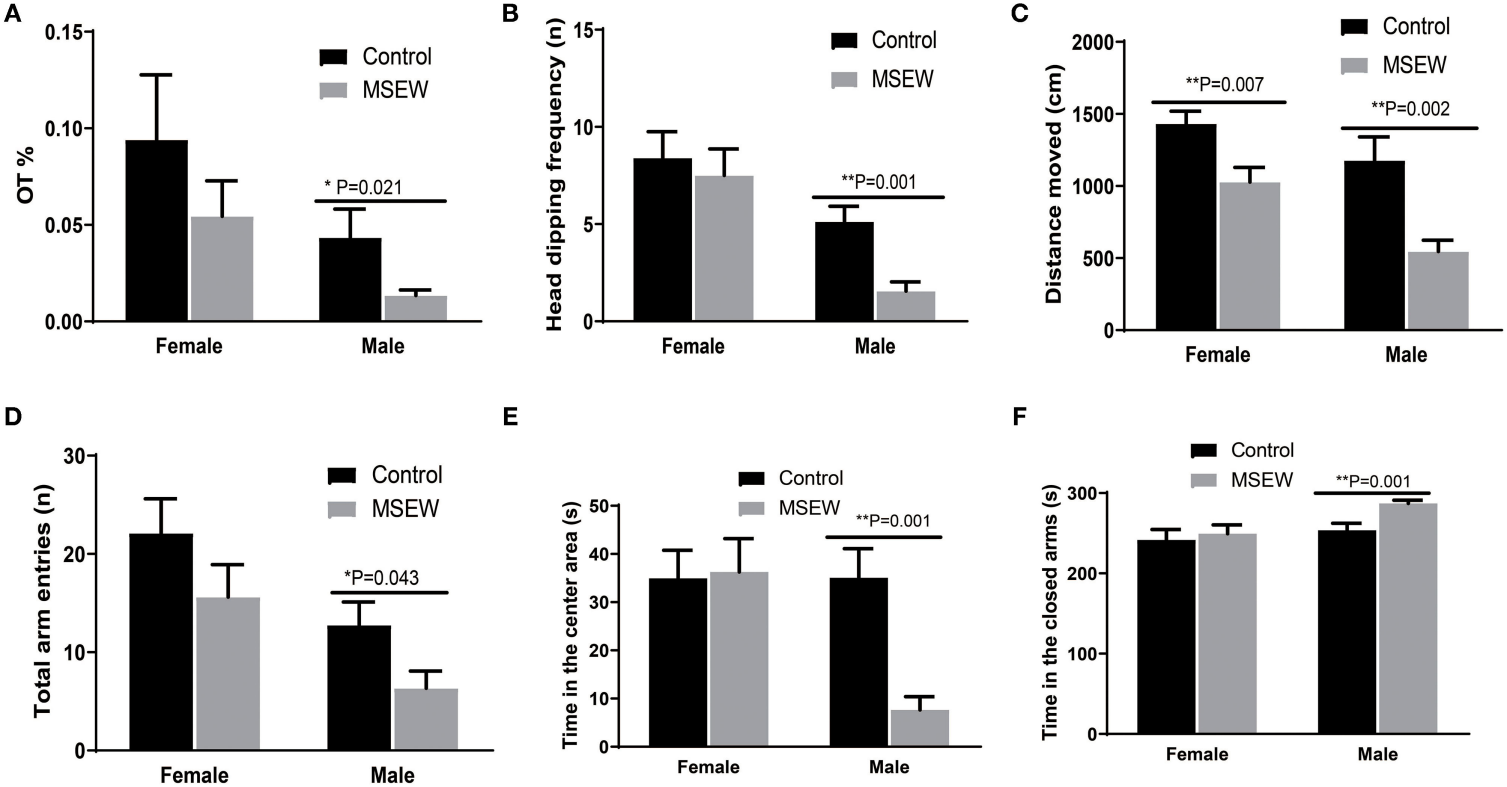
Maternal separation with early weaning (MSEW) induced anxiety-like behavior in the elevated plus maze test in male rats and decreased locomotor activity in both sexes. Male MSEW rats exhibited reduced percentage of time spent on the open arms (%OT), head-dipping frequency, distance moved, and total entries **(A–D)**. MSEW reduced the time in the center area and increased the time in the closed arms in male rats **(E,F)**. All results are presented as the mean ± standard error of the mean (SEM) of *n* = 8–10 rats per group. **P < 0.01, control vs. MSEW of the same sex; *P < 0.05, **P < 0.01, control vs. MSEW of the same sex.

There was no interaction of sex and MSEW in the percentage of time spent on the open arms (%OT) [*F*_(3.33)_ = 0.05, *P* = 0.824], head-dipping frequency [*F*_(3.33)_ = 1.397, *P* = 0.246], distance moved [*F*_(3.33)_ = 1.106, *P* = 0.301], time in the closed time [*F*_(3.33)_ = 1.593, *P* = 0.216], or total entries [*F*_(3.33)_ = 0.209, *P* = 0.989]. Significant main effects of MSEW and sex were found on head-dipping frequency [*F*_(3.33)_ = 3.916, *P* = 0.050; *F*_(3.33)_ = 16.603, *P* = 0.000] and distance moved [*F*_(3.33)_ = 23.117, *P* = 0.000; *F*_(3.33)_ = 11.654, *P* = 0.002]. The assumption of normality for %OT was not met (*P* = 0.023), and a Mann–Whitney test was conducted. MSEW reduced %OT, head-dipping frequency, distance moved, and total entries in male rats (Figures 2A–D). A sex × MSEW interaction for the time in the center was observed [*F*_(3.33)_ = 6.306, *P* = 0.017]. Significant main effects of MSEW and sex were found on the time in the center [*F*_(3.33)_ = 5.170, *P* = 0.030; *F*_(3.33)_ = 6.171, *P* = 0.018] and the time in the closed arms [*F*_(3.33)_ = 4.051, *P* = 0.052; *F*_(3.33)_ = 6.306, *P* = 0.020]. MSEW reduced the time in the center area and increased the time in the closed arms in male rats (Figures 2E,F).

### Effect of Maternal Separation With Early Weaning on Memory

#### Novel Object Recognition Test

**Figure 3:**
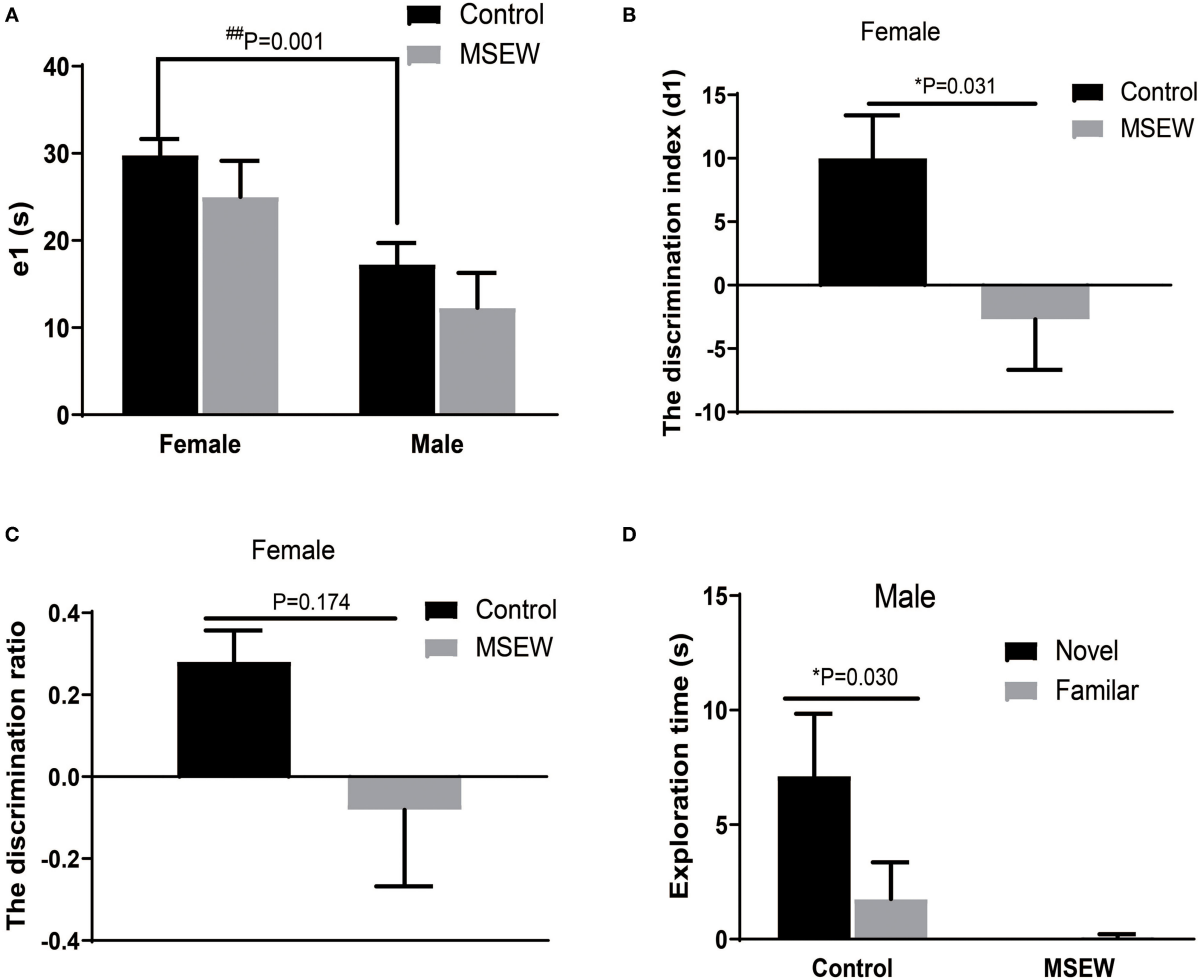
Maternal separation with early weaning (MSEW) slightly impaired non-spatial working memory in the novel object recognition test in female rats. The total exploration time did not differ between the control and MSEW groups of the same sex, and female control rats showed a longer exploration time than male control rats **(A)**. During the test session (2 h later), female MSEW rats showed significantly reduced discrimination index (d1) but no change in the d1 compared to the control females **(B,C)**. Male MSEW rats showed very low exploration levels of both the novel object and the familiar object **(D)**. All results are presented as the mean ± standard error of the mean (SEM) of *n* = 8–10 rats per group. *P < 0.05, control vs. MSEW of the same sex; ^##^*P* < 0.01, male vs. female in controls.

During the training session, there was no interaction of sex and MSEW in the total exploration time (e1) [*F*_(3.33)_ = 0.01, *P* = 0.978]. A significant main effect of sex was found [*F*_(3.33)_ = 14.638, *P* = 0.01]. The total exploration time did not differ between the control and MSEW groups of the same sex (Figure 3A). Exploration time was longer in control female rats than in control male rats (Figure 3A). The total exploration time of control male rats did not reach 20 s. During the test session (2 h later), MSEW female rats had a significantly reduced d1, but their discrimination ratio did not change compared to that of the control females (Figures 3B,C). MSEW male rats showed a very low exploration level of both the novel object and the familiar object (Figure 3D).

#### Y Maze Novel Preference Arm Test

**Figure 4:**
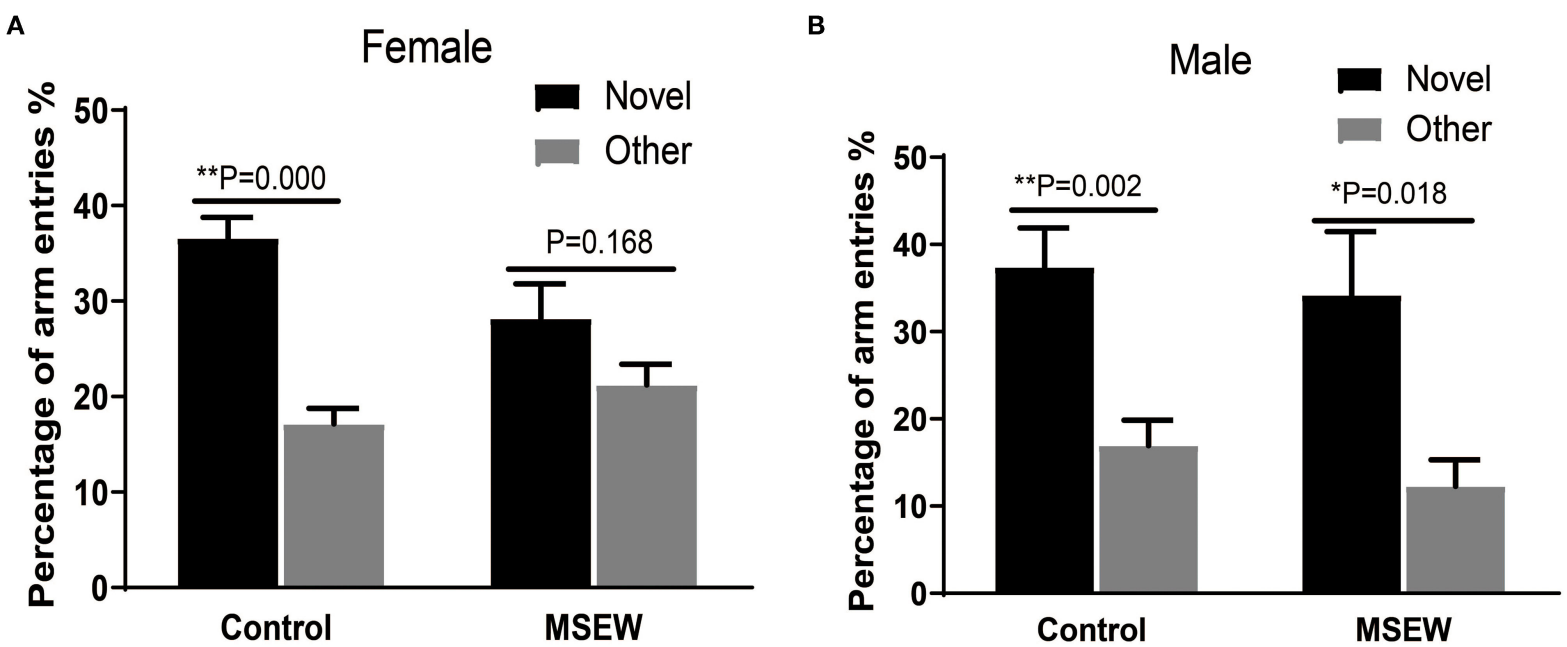
Maternal separation with early weaning (MSEW) slightly impaired spatial working memory in the Y maze in female rats. Female control rats entered the novel arm more than the other arm **(A)**. Female MSEW rats also showed a preference for the novel arm, but the difference was not significant **(A)**. Both control and MSEW male rats entered the novel arm more than the other arm **(B)**. All results are presented as the mean ± standard error of the mean (SEM) of *n* = 8–10 rats per group. *P < 0.05, **P < 0.01, control vs. MSEW of the same sex.

The 1st min that contained the greatest locomotor activity was investigated. There was no interaction of sex and MSEW in the discrimination preference in the 1st min [*F*_(3.33)_ = 1.943, *P* = 0.173]. Significant main effects of MSEW and sex were also not found [*F*_(3.33)_ = 1.230, *P* = 0.275; *F*_(3.33)_ = 2.565, *P* = 0.119]. Control female rats entered the novel arm more than the other arm (Figure 4A). MSEW female rats showed a preference for the novel arm (Figure 4A). Both control male rats and MSEW male rats entered the novel arm more than the other arm (Figure 4B).

### Effect of Maternal Separation With Early Weaning on Spatial Reference Memory

#### Barnes Maze Test

**Figure 5:**
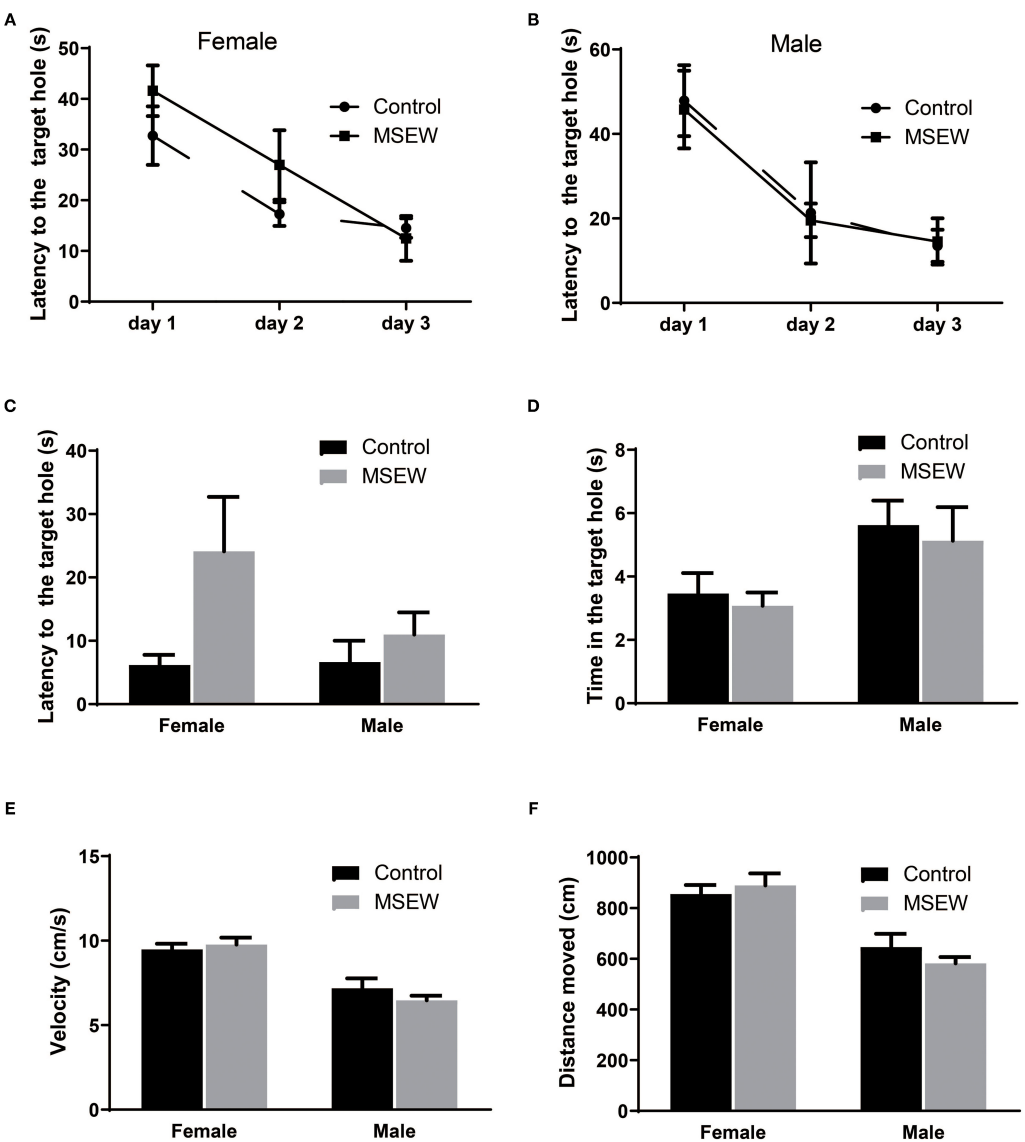
Maternal separation with early weaning (MSEW) did not impair spatial reference memory in either sex. Compared with control females, MSEW female rats had a tendency to spend more time finding the platform (latency) on the 1st and 2nd days and spent similar time on the 3rd day **(A)**. MSEW and control male rats spent similar amounts of time finding the platform **(B)**. For the probe test, MSEW rats showed no significant difference compared to their respective controls in latency to the target hole, time to the target hole, velocity, or distance moved for either sex **(C–F)**. All results are presented as the mean ± standard error of the mean (SEM) of *n* = 8–10 rats per group.

In the training phase, for the latency to find the target hole over the 3 days of training, there was no effect of a treatment × days interaction [*F*_(3.33)_ = 0.781, *P* = 0.513]. There was an effect of days [*F*_(3.33)_ = 49.561, *P* = 0.000] but no effect of treatment [*F*_(3.33)_ = 0.452, *P* = 0.711]. Three-way ANOVA analysis showed that significant main effect of sex was not found [*F*_(3.33)_ = 0.439, *P* = 0.512]. Compared to control female rats, MSEW female rats had a tendency to spend more time finding the platform on the 1st and 2nd days but similar time on the 3rd day (Figure 5A). MSEW and control male rats spent similar time finding the platform (Figure 5B). For the probe test, there was no interaction of sex and MSEW in the latency to find the escape box [*F*_(3.33)_ = 1.722, *P* = 0.199]. No significant differences between MSEW rats and their respective controls were found for latency to the target hole, time in the target hole, velocity, or distance moved in either sex (Figures 5C–F).

### Effect of Maternal Separation With Early Weaning on Coping Behavior

#### Forced Swim Test

**Figure 6:**
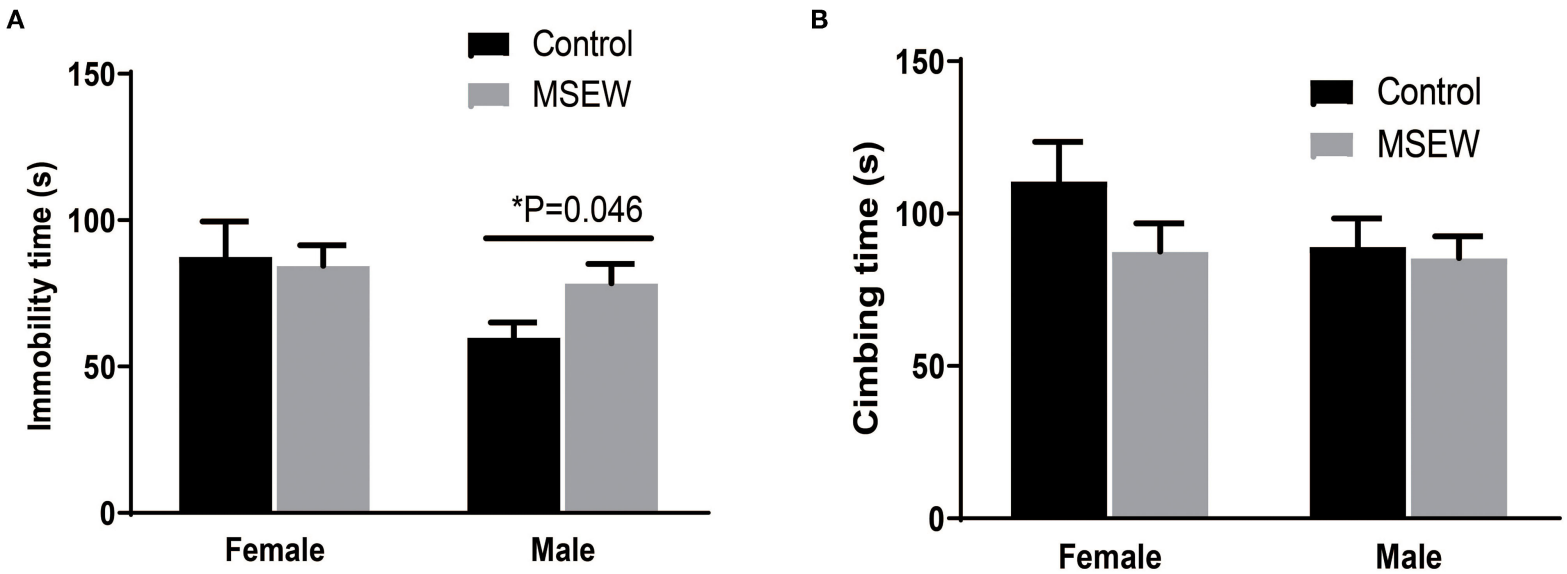
Maternal separation with early weaning (MSEW) induced a passive coping strategy in male rats. MSEW increased the immobility time in males. **(A)** MSEW did not change the climbing time in either sex. **(B)** All results are presented as the mean ± the standard error of the mean (SEM) of *n* = 8–10 rats per group. *P < 0.05, control vs. MSEW of the same sex.

There was no interaction of sex and MSEW in the immobility time [*F*_(3.33)_ = 1.628, *P* = 0.211] or climbing time [*F*_(3.33)_ = 0.923, *P* = 0.344]. A trend-level effect of sex was found [*F*_(3.33)_ = 0.295, *P* = 0.057]. MSEW increased the immobility time in males (Figure 6A). MSEW did not change the climbing time in either sex (Figure 6B).

### Effect of Maternal Separation With Early Weaning on Fear Memory

#### Fear Conditioning Training

**Figure 7:**
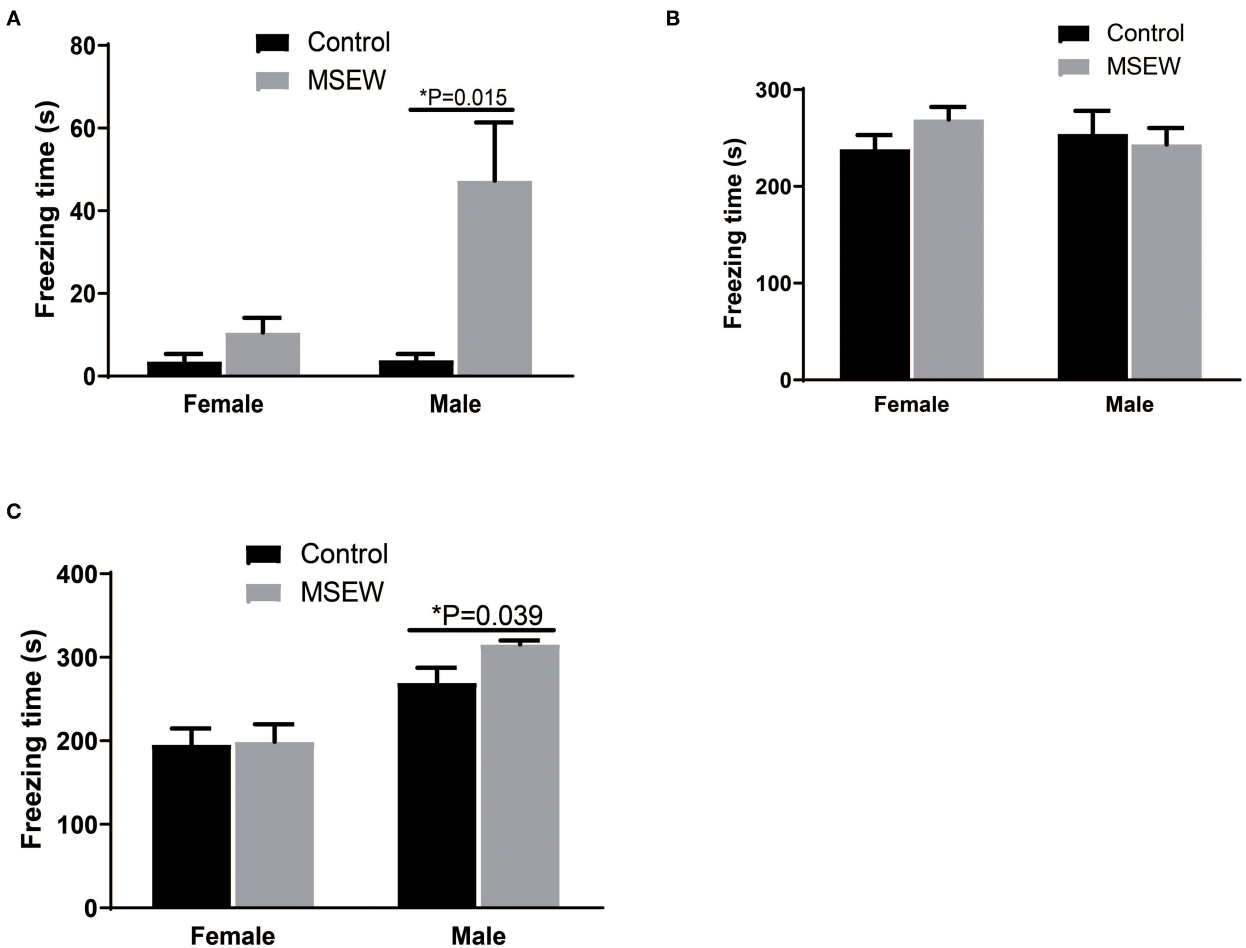
Maternal separation with early weaning (MSEW) did not impair associative learning in either sex or increase fear memory in male rats. MSEW male rats showed more freezing behavior than control male rats **(A)**. Female and male MSEW rats did not show impaired associative learning **(B)**. MSEW male rats exhibited increased freezing time compared to the control male rats **(C)**. All results are presented as the mean ± the standard error of the mean (SEM) of *n* = 8–10 rats per group. *P < 0.05, control vs. MSEW of the same sex.

In the first 3-min habituation, there was an interaction of sex and MSEW in the freezing time [*F*_(3.33)_ = 6.359, *P* = 0.017]. Significant main effects of MSEW and sex were found on the baseline freezing time [*F*_(3.33)_ = 11.218, *P* = 0.002; *F*_(3.33)_ = 6.146, *P* = 0.018]. The MSEW male rats showed more freezing behavior than the control male rats (Figure 7A). In the fear conditioning training phase, there was no interaction effect of sex and MSEW in the freezing time [*F*_(3.33)_ = 1.467, *P* = 0.234]. Significant main effects of MSEW and sex were also not found [*F*_(3.33)_ = 0.345, *P* = 0.561; *F*_(3.33)_ = 0.089, *P* = 0.767]. Female and male MSEW rats did not demonstrate impaired associative learning (Figure 7B).

#### Contextual Fear Conditioning

There was no interaction of sex and MSEW in the freezing time in the contextual fear conditioning test [*F*_(3.33)_ = 1.460, *P* = 0.235]. A significant sex effect was found [*F*_(3.33)_ = 0295, *P* = 0.000]. No significant main effects of MSEW were found [*F*_(3.33)_ = 1.985, *P* = 0.168]. MSEW increased freezing time in male rats (Figure 7C).

### Effect of Maternal Separation With Early Weaning on Neuronal Excitability

**Figure 8:**
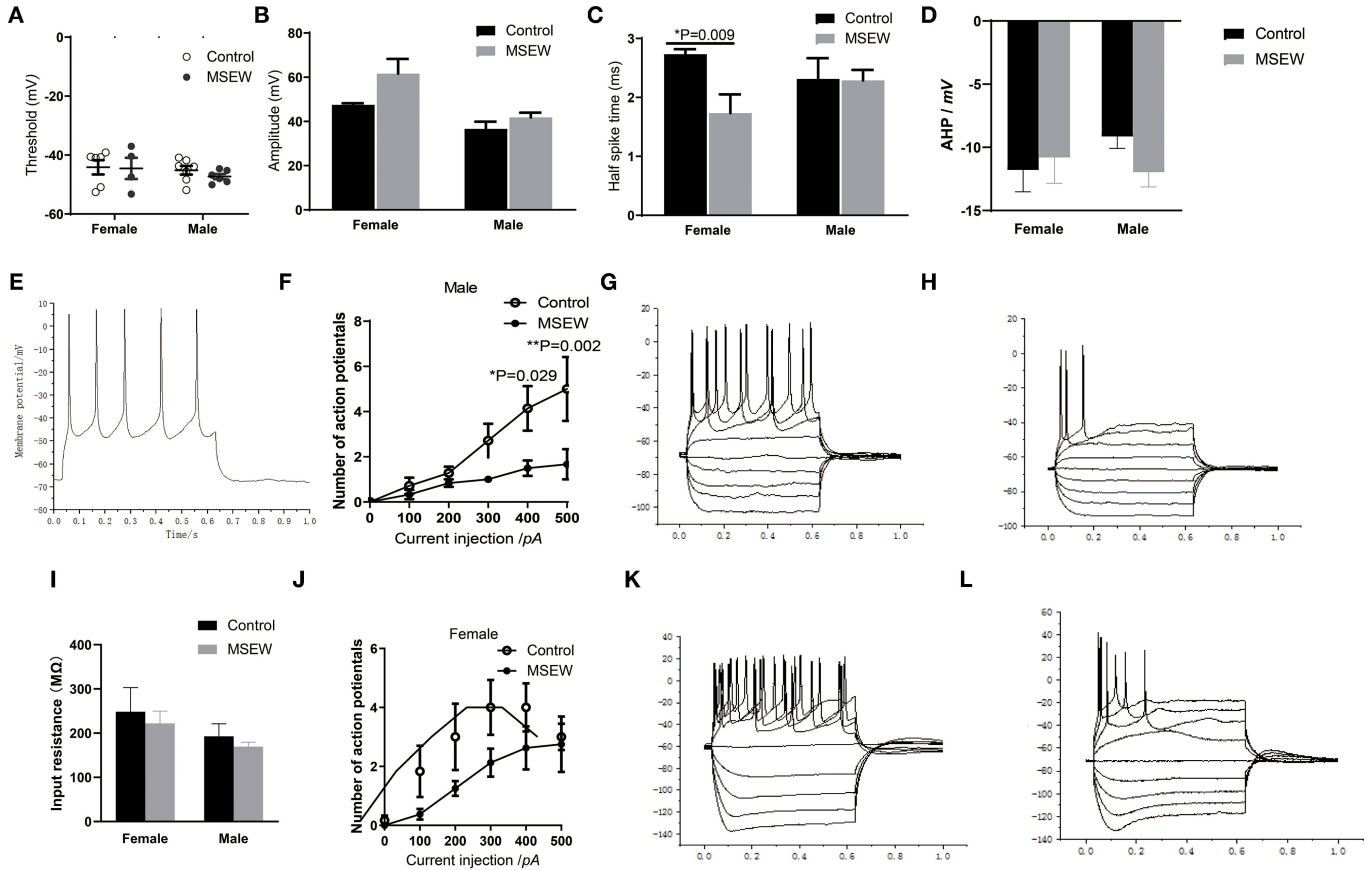
Maternal separation with early weaning (MSEW) reduced glutamatergic neuronal excitability in the prefrontal cortex in male rats. MSEW did not change the threshold or amplitude of single action potentials in the prefrontal cortex in either male or female rats **(A,B)**. MSEW decreased the half-spike time in female rats **(C)**. Afterhyperpolarization (AHP) in the four groups **(D)**. Representative traces of glutamatergic neurons (150 pA, 600 ms) **(E)**. MSEW male rats had decreased numbers of glutamatergic neuron action potentials (APs) with current injections of 400 and 500 pA **(F)**. Representative traces obtained in the prefrontal cortex neurons from control and MSEW male rats **(G,H)**. Input resistance in the four groups **(I)**. MSEW female rats showed a trend for decreased numbers of glutamatergic neuron APs **(J)**. Representive traces obtained in the prefrontal cortex neurons from control and MSEW female rats **(K,L)**. All results are presented as the mean ± standard error of the mean (SEM) of *n* = 3–5 rats per group. *P < 0.05, **P < 0.01, control vs. MSEW of the same sex.

MSEW did not change the threshold or amplitude of single APs in the PFC in either sex (Figures 8A,B). MSEW decreased the half-spike time in female rats (Figure 8C). AHP for each group was presented and input (Figure 8D). Representative traces of glutamatergic neurons were presented (Figure 8E). With 400 and 500 pA current injections, MSEW male rats exhibited significantly decreased numbers of glutamatergic neuron APs compared to control males (Figure 8F). Representative traces obtained in the prefrontal cortex neurons from control and MSEW male rats were presented (Figures 8G,H). Input resistance for each group was presented (Figure 8I). MSEW female rats showed a trend for decreased numbers of glutamatergic neuron APs (Figure 8J). Representative traces obtained in the prefrontal cortex neurons from control and MSEW female rats were presented (Figures 8K,L).

## Discussion

Despite an extensive body of literature, there is no consensus on the behavioral effects induced by MS and the extent of these effects. A longer MS period was used to reduce any potential for compensatory maternal care after MS. In addition, a previous study indicated that the early-life stress phenotype was strongest when early-life stress was combined with other negative experiences. In our study, we observed a longer MS period with early weaning-induced long-lasting behavioral changes and electrophysiological alterations in the PFC that persisted into adulthood.

### Maternal Separation With Early Weaning Induced Anxiety-Like Behavior and Passive Coping Strategy in Male Rats

In preclinical research, the open field test and elevated plus maze test are widely used to assess anxiety-like behavior in rodent animals (26, 27). In our study, only male MSEW rats showed an anxiogenic phenotype with decreased central activity in the open field test, as well as decreased time in the open arms and head-dipping frequency in the elevated plus maze test, which is in agreement with previous work showing strong sex-dependent bias on anxiety behavior with males (28–31). Few studies have reported that MS induces anxiety behavior in female animals (32). Notably, in our subsequent study, we found that MSEW also induced anxiety-like behavior in adolescent SD rats (data not shown). Taken together, our findings suggested that MSEW induced a sustained anxiety phenotype in male rats. Conversely, in the clinic, women are twice as likely as men to exhibit anxiety disorders and are believed to possess an innate vulnerability that makes them susceptible to anxiety disorders (33). The difference in developmental timing between rodents and humans may be relevant to the discrepancy. Another possibility is that the method of what is detected in rodents does not accurately reflect the human condition (34).

Similar to the anxiety phenotype, inconsistent behavior results for depressive-like behavior were reported for MS in rats (35–37). Our results showed that MSEW induced a passive coping strategy with increased immobility time in the forced swim test in male rats. More behavioral tests, such as anhedonic assessments, are needed to identify the depressive-like behavior induced by MSEW.

### Maternal Separation With Early Weaning Induced Decreased Locomotor Activity in Both Sexes

In the present study, MSEW induced decreased locomotor activity in the open field test, elevated plus maze test, and Y maze test in both sexes. MS induced paradoxical behavior results for the locomotor activity effect. MS on PNDs 3–14 and on PNDs 3–21 did not affect locomotor activity in Long-Evans rats (31). A single 24-h period of maternal deprivation on PND 9 resulted in a decrease in locomotion in Wistar rats (38). MSEW caused hyperactivity in the open field test in mice (16). Different methodologies of MS may lead to these inconsistent results. Interestingly, our findings showed that MSEW did not affect locomotor activity in either sex in the Barnes maze test. Notably, the open field test, elevated plus maze test, and Y maze test are based on the spontaneous exploratory behavior of rodents, while the Barnes maze test is based on negative reinforcement of behavior (bright lights and loud buzzing). A previous study reported that MS may improve stress coping in adult rats, reflected by increased offensive-like behavior during juvenile play-fighting and aggression resident–intruder tests (39, 40). As such, our results suggested that negative reinforcement in the Barnes maze test increased the locomotor activity level of MSEW rats and, to some extent, improved their stress coping in a stressful circumstance. In addition, our study indicated that female rats showed higher levels of exploration and locomotion than male rats, as measured by a significant increase in distance moved and velocity in the open field test and open arm entries in the elevated plus maze, as well as total arm entries in the Y maze and exploration time of the familiar object in the NOR test. These findings supported previous work that reported that female SD rats exhibited more curiosity and showed greater locomotion than males in social and non-social behavioral tests (41).

### Maternal Separation With Early Weaning Slightly Impaired Working Memory in Female Rats but Did Not Impair Spatial Reference Memory in Either Sex

Working memory in humans is considered a distinct short-term memory process. Working memory reflects the capacity to temporarily maintain and manipulate recently acquired information actively for further goal-directed actions (42). Working memory is impaired in some neurodegenerative diseases (43) and most psychiatric and developmental disorders, such as schizophrenia and attention-deficit hyperactivity disorder (44). Clinical practice has reported that early-life stress is related to working memory deficits (45). Spatial spontaneous alternation behavior in the Y maze has been viewed as a test of spatial working memory in rodents that requires active maintenance and the manipulation of information in a limited time (46). We used the Y maze delay spontaneous alternation protocol to determine the effects of MSEW on spatial working memory. Our results showed that MSEW female rats, but not MSEW male rats, displayed a lower preference for the novel arm than control females in the 1st min, which indicated that MSEW led to deficits in spatial working memory in female rats. The sex-dependent bias on working memory deficits in females in our study differs from those of some studies that reported that working memory was also impaired in males (47, 48). We did not find MSEW-induced deficits in recognition memory in the Y maze novel preference arm test in male rats.

The NOR test is a simple and sensitive behavioral assay for the evaluation of non-spatial memory. In the present study, after a 2-h retention delay, our results showed that MSEW decreased the discrimination index in female rats, which indicated that MSEW impaired non-spatial memory in female rats. Statistically significant differences were not found between control and MSEW female rats in the discrimination ratio. We could not estimate whether MSEW impaired the recognition ability in male rats due to their low level of exploration.

In the present study, the Barnes maze test was used to detect the effect of MSEW on spatial reference memory. Our results showed that MSEW did not impair learning in the training phase or spatial memory in the probe test in either sex, although MSEW had a tendency to impair learning on the 1st and 2nd days in the training phase in females. MS resulted in inconsistent behavior in the spatial reference test. This result is in line with previous studies that have reported that MS (10 and 21 days) did not alter spatial long-term memory in the Morris water maze test (a similar paradigm to the Barnes maze test for evaluating spatial reference memory) in female or male Wistar rats (49, 50).

### Maternal Separation With Early Weaning Did Not Impair Associative Learning in Either Sex but Increase Fear Memory in Male Rats

In the habituation phase of the fear conditioning test, MSEW male but not female rats showed more anxious behavior, measured by greater freezing behavior, than control male rats. This result is consistent with our findings in the open field test and elevated plus maze test, which also showed that MSEW induced anxiety-like behavior in male rats. In the fear conditioning training phase, MSEW did not impair fear learning in either sex. In the contextual fear conditioning test, MSEW male rats exhibited increased fear memory. No effect was observed in females. Our current finding concurred with previous work that suggested that anxious rats exhibited greater fear memory (51). Contextual fear conditioning is hippocampus-dependent. Further research is needed to investigate the role of the hippocampus in the effect of early-life stress on anxiety-like behavior and fear memory.

Notably, the NOR test and Y maze test are based on the spontaneous exploratory behavior of rodents and are non-stressful, while the Barnes maze test and fear conditioning test are based on stressful situations. Bonapersona et al. (30) used a large-scale three-level meta-analysis of all peer-reviewed preclinical literature and provided extensive evidence that early-life stress impaired non-stressful learning and enhanced memory formation during stressful learning. In the current study, MSEW increased locomotor activity and improved stress coping in stressful situations, which suggested that MSEW induced adaptive modification in stressful situations. MSEW female rats showed slight working memory deficits in the novel recognition test and Y maze test and a trend-level effect on spatial learning impairment in the Barnes maze, which supports theories in which there is a close link between working memory mechanisms and long-term memory mechanisms (52).

### Maternal Separation With Early Weaning Reduced Glutamatergic Neuronal Excitability in the Prefrontal Cortex in Male Rats

Neural intrinsic excitability determines the net output of neurons by integrating synaptic inputs and consecutively translating them into AP firing (20). Our study demonstrated that MSEW reduced glutamatergic neuronal excitability significantly in the PFC in male rats, as shown by decreased numbers of glutamatergic neuron APs upon current injection, although the parameters of single APs did not change. Neuronal intrinsic excitability reflects global changes (53). Many voltage-gated and ion channels are implicated in shaping the spiking output (20). Further research is needed to identify the mechanism by which MSEW affects glutamatergic neuronal excitability. The reduced glutamatergic neuronal excitability may be associated with the emotional alteration induced by MSEW in male rats (54, 55). Although MSEW reduced the half-spike time, we could not infer the effect of MSEW on glutamatergic neuron single APs in female rats. The effect of MSEW on the glutamatergic neuronal excitability and its relationship with the damage of working memory in female rats need further study.

There is a limitation to this study. Growing evidence has shown that sex hormone status plays an important role in modulating rodent behavior (56). In the current study, the estrous cycle stage was not recorded, and the effect of sex hormones on the behavior of female rats was ignored. In addition, previous reports have shown that the female estrous cycle also induced I sex differences in neuron excitability and behavior (57, 58). Hippocampal excitability is more variable at proestrous (59). Our results showed a high level of variability in the number of APs in female rats across a range of current steps, which may be associated with female estrous cycle effects. MSEW affects different aspects of glutamate neuron properties in males and females, which may be partly linked to the estrogen cycle (60). Collectively, these data provide the first evidence that early-life stress modulates the intrinsic excitability of glutamatergic neurons in the PFC.

## Conclusion

In summary, we combined MSEW to create an early-life neglect rat model, inducing long-lasting anxiety-like behavior in male rats, to unravel possible influences of early-life stress on adult anxiety disorder and screen for potential therapies. The reduced glutamatergic neuronal excitability may be associated with the emotional alteration induced by MSEW in male rats. MSEW affected memory formation in a sex-dependent manner. In addition, MSEW slightly impaired memory during non-stressful situations and did not change learning or memory under stressful circumstances. This finding suggested that child neglect induced an adaptive modification only in a stressful context.

## Data Availability Statement

The datasets generated for this study are available on request to any qualified researcher.

## Ethics Statement

The animal study was reviewed and approved by the ethical committee for the use of experimental animals of the Institute of Laboratory Animal Sciences.

## Author Contributions

CQ conceived, designed the experiments, and acquired the funding. XS designed, performed the behavioral tests, and wrote the manuscript. YZ performed the electrophysiological tests. XLi performed the behavioral tests. XLiu conceived the experiments and acquired the funding. All authors contributed to the article and approved the submitted version.

## Conflict of Interest

The authors declare that the research was conducted in the absence of any commercial or financial relationships that could be construed as a potential conflict of interest.

## Abbreviations

MSEW: maternal separation with early weaning
MS: maternal separation
AP: action potential.
